# A Chair of Medical Ethics

**Published:** 1917-02

**Authors:** 


### A Chair of Medical Ethics.

It is occasionally suggested that medical colleges, at least those of more generous endowment, should establish a professorship to instruct students in medical ethics. At first thought, it would seem quite unnecessary to give formal instruction as to good conduct and courtesy yet it must be remembered that there are a good many quite complicated problems which the young man, without much experience or tact and with views based on those of a lay family, often does not adequately comprehend. Some physicians, indeed, go through life, making mistakes not at all chargeable to improper motives. On the other hand, a chair of ethics may be considered somewhat analogous to a chair of morals in a literary college, it would be likely to excite ridicule or further distrust among the laity and there is danger that a man, however conscientious, formally engaged to specialize in ethics, might conceive it to be his duty to imitate the research work in more practical specialties and might present to students, a too elaborate course including recondite methods of dealing with imaginary evils, and a formal and inelastic system of etiquette.

As medical ethics is something to be used along with actual practice, it seems that it would be better to have it presented to students incidentally, by the same men whom he respects as scientific and practical teachers. For instance, at the beginning of clinic sessions, the general principle of privileged communications and professional respect for the confidences of patients, should be put clearly before students; the practical exception necessary for their instruction should be emphasized as an exception; and it should be impressed on the students that each is, in this respect, a consultant, free to discuss with the other consultants, the scientific and clinical features of the case but bound to refrain from prying into purely personal affairs and from communicating any information to outsiders. Possibly by exaggerating somewhat the deference due to physicians bringing in cases, by insisting on their examining or reporting on their previous examinations of the case, before the case is examined by the clinical lecturer, the mutual relations of attendant and consultant will themselves be taught in clinical form. Even without much formal division of the work among the faculty, the two years in which clinical demonstrations occupy a considerable part of undergraduate courses would be ample to impress on students, by repetition and view from different angles, practically all the ethical problems with which they will be confronted. Nearly every graduate now has an interne service. Equal care to show to and to demand from the interne, the practice of ethics and even of etiquette would firmly clinch this teaching and breaches of ethics would be perpetrated only by those so constituted that they would violate the standards of any profession or business. Occasionally a physician, not regularly attending a hospital is offended at the treatment received from an interne and surprised at the stupidity of one who will shortly be dependent to a large degree on the good will that he creates. In many cases of this sort, the interne has been so thoroughly kicked about by those in authority over him that he may even consider this method the proper expression of his own authority. In other cases, he is acting through a mistaken sense of loyalty, sometimes apparently encouraged by his superiors, sometimes due to their neglect to exercise the guidance over the young men in their charge.

As medical colleges approach the ideals and methods of more strictly and generally educational institutions, it becomes possible for them to adopt the assembly of the student body before the work of the day begins, for devotional exercises and for the inculcation of general principles of right which in the chapel talks of schools and colleges are sometimes tedious but which ultimately exert a great influence for good over the whole after life of the students. Regular meetings of this sort, would develop a sentiment of unity and loyalty among students and would afford abundant opportunity for the establishment of high professional ideals and even for detailed consideration of the principles of ethics by which the profession is guided.

